# Reducing the mechanical wear of elbows and pipes due to solid particles flow by using nano coating technique

**DOI:** 10.1038/s41598-021-01563-1

**Published:** 2021-11-15

**Authors:** Ali Sadiq Al-Ithari, Nabeel Al-Zurfi, Laith Zbbal Abd U. L. Kareem

**Affiliations:** 1 Department of Mechanical Engineering, University of Kufa, University of Kufa, P.O.Box (21), Najaf Governorate, Iraq; 2 Ministry of Industry and Minerals /Company of Iraqi Cement/Najaf Cement Plant, Najaf, Kufa, P.O.(25) Najaf Governorate, Iraq

**Keywords:** Nanoscale materials, Mechanical engineering

## Abstract

This work investigated reasons and factors that cause the failure due to mechanical wear (Erosion) for the inside surface of elbows and pipes used in cement transportation which manufactures from low carbon steel and finds out a method for reducing this failure. The technique of Nano-coating layers is used to coat the surface of samples with layers of nanoparticles of tungsten carbides of different thicknesses of (30, 40, and 50 μm). The test was done for these samples by placing them inside the elbow under the same operating conditions, pin on disc test. The results of the test under the same operation condition showed a decrease in erosion rate by 71% for the sample coated with 50 μm of layer, while the results of the pin on disc test showed a decrease in erosion rate by 97% for the thickness of 50 μm as this test is done under ideal testing conditions. The decrease in wear rate for elbow and pipes will increase their life work two times at least and that reduces the cost of maintenance by about 75%. The numerical simulation was also implemented to simulate the erosion profile inside the elbow, and the agreement with experimental results was 90%.

## Introduction

The term slurry defines as a "Non-homogenous mixture of solid with liquid, gas or air". The flow of cement and high-speed air mixture may cause mechanical wear for the internal surface of the pipes and elbows and that leads to big losses in money and time. The particle sizes of a solid vary from few microns to few millimeters. The effect of a high particle concentration is influenced by different factors such as density amount, size, mass part, particles of solid and carrier density; and mortar can be classified as an unstable factor depending on the size of the solid particle. The ability of solid particles to stabilize the carrier air is low^1^. Many fine powders, such as cement, which exhibit very low de-aeration rates are suitable for dense-phase transport. Observation of the flow patterns, in horizontal pipes, when these powders are transported in dense phase reveals a stratified flow. A high concentration layer of fluidized material occupies the lower portion of the pipe. In the upper portion of the pipe, particles are suspended in the transport gas. The fluidized layer generated at the top and bottom of the pipes makes the particles suspended in the transport air^2^. Pipelines are important for transporting gases, liquids, and solid materials for long distances from their main source to warehouses or silos. The cement industry is one of the important industries in the world, as it is involved in a lot of applications in building and construction^3^. The erosion-corrosion rate investigated 30°, 60°, and 90° carbon steel elbows in a multiphase flow containing sand particles. Qualitative techniques such as multilayer paint modeling and microscopic surface imaging were used to scrutinize the flow accelerated erosion-corrosion mechanism. The results show the erosion rate for the elbow of 90° higher than in low angle elbows (30° and 60°), also the high erosion rate occurs in the upper half of the elbow^4^. The transport of these conditions at high pressures up to 7 bar where the flow is turbulent and the particles of cement distributed directly across the cross-section of the pipe because of the high level of turbulence. Solid particles have a high impact on metal surfaces; causing damage to the surface and remove parts from it. This phenomenon is called mechanical wear (Erosion). Erosion always occurs when the solid particles directly hit a solid surface. The mechanical erosion (wear) inside pipes plays a crucial role in the design and operation of the transporting system^5^. Investigations of wear are done for areas of interest of different industries. Earlier efforts in the field of corrosion research began in 1960 in the industrial countries; this shows the complications of the wear phenomenon^6^. The mild steel used to study the wear occurred in pipes, where a mixture of compressed air and abrasive solid particles was made. Their results indicated that the decrease in the bend curve leads to a decrease in wear^7^. The erosion in mud transport tubes using ANSYS software was studied numerically; the results showed that the erosion rate increases eight times^8^. The imperative used to simulate Nanoindentation by powerful FEM software to extract plenty of mechanical properties like hardness, elastic modulus, endurance loads and various parameters like optimal thickness and optimal critical load, stress distribution and contact pressure between substrate and layer can be obtained through load–displacement curve A^9^. The wear resistance and scratch were investigated to St-37 mild steel after coating it with a nanoparticle of nickel-boron. The method used is electrical deposits and heat treatment with a period of (1 h) at a temperature of (400 °C); the results showed a decrease in the wear rate of about 50%, while hardness increased by about 28%^10^. An experimental study on pipelines was conducted to find out the reasons that lead to erosion by using a sandblasting device for different values of flow speed (20–80 m/s). The results showed that at a high speed and for a long time, the erosion was an abrasive mechanism, whereas at a low speed the erosion was due to plastic deformation^11^. A numerical study on mild steel was conducted to find the wear. A steel pipe of 1.5 m in length and 50–250 mm diameter is used in their study. The sand is mixed with water to form slurry and to control the speed from within the range of 2–8 m/s. The results showed that the greater the bending angle, the greater the wear. The largest wear ratio was at an angle of 30°–60° degrees^12^.The Nano coating layer of Tungsten Carbide is used to coat the surface of high chromium cast iron by using a thermal spray process and studied the improvement in the wear resistance and microhardness. The result showed that the microhardness increased by a ratio of 34% and wear resistance increased by 79% due to the good wear resistance properties for Nanopowder of tungsten carbide and good adhesion properties with steel surfaces at high temperature in the thermal spray process^13,14^. It appears from the aforementioned literature review and to the best of the Authors’ knowledge, the study of the mechanical wear caused by cement particles passing through a 90° degree pipe elbow with a unique method to reducing this mechanical wear by coating the internal surface of the elbow with layers of nanoparticles of tungsten carbides (WC) has not been studied extensively yet. The objective of the present work is to find the technique to reduce the wear rate of elbow and pipes to increasing their work life and reducing the maintenance cost and reducing the production stopped. The experimental and numerical study would be a considerable contribution to the open literature and conducted the improvement in wear resistance for coated inner the surface of elbows and pipes that used in pneumatic conveyors for cement transportation.

## Experimental work

### The used materials

The metals of low carbon steel are used in the manufacturing of pipes and elbows in most of the cement plants. The chemical composition of low carbon steel listed in Table1. This material is also used in the preparation of samples tested inside elbow; pin on the disc, hardness test, and SEM image analysis.

**Table 1:** Chemical composition of low carbon steel.

Composition	C	Si	Mn	P	S	Cr	Mo	Ni	Al	Cu	Fe
Experimental Wt. %	0.214	0.257	0.808	0.0101	0.0007	0.083	0.0034	0.0968	0.0418	0.176	Bal

### The coating material

**Figure 1:**
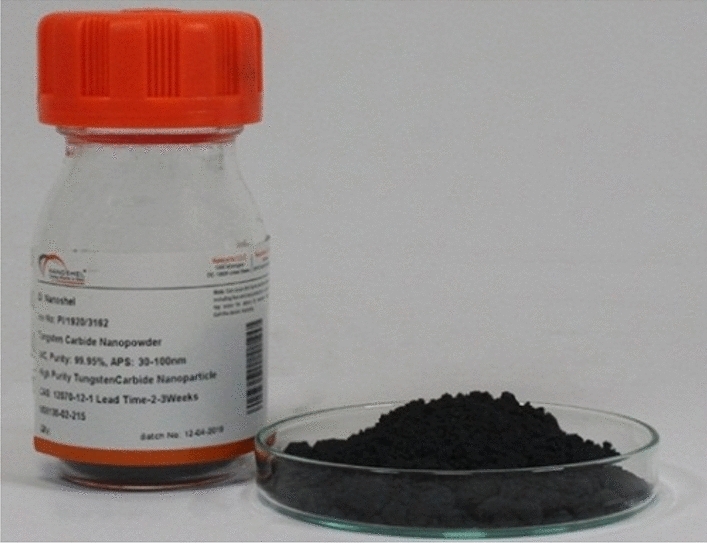
Nanoparticles of tungsten carbide (WC)^13^.

The Nano particles of tungsten carbide (WC) has Nano-size of (55 nm) used as a coating material to coat the low carbon steel samples. This nanoparticles has true density of 15,500 kg/m^3^, melting point of 2870 °C, boiling point of 6000 °C. Figure 1 shows the nanoparticles of tungsten carbides that used as coating materials.

### Thermal spray technique

**Figure 2:**
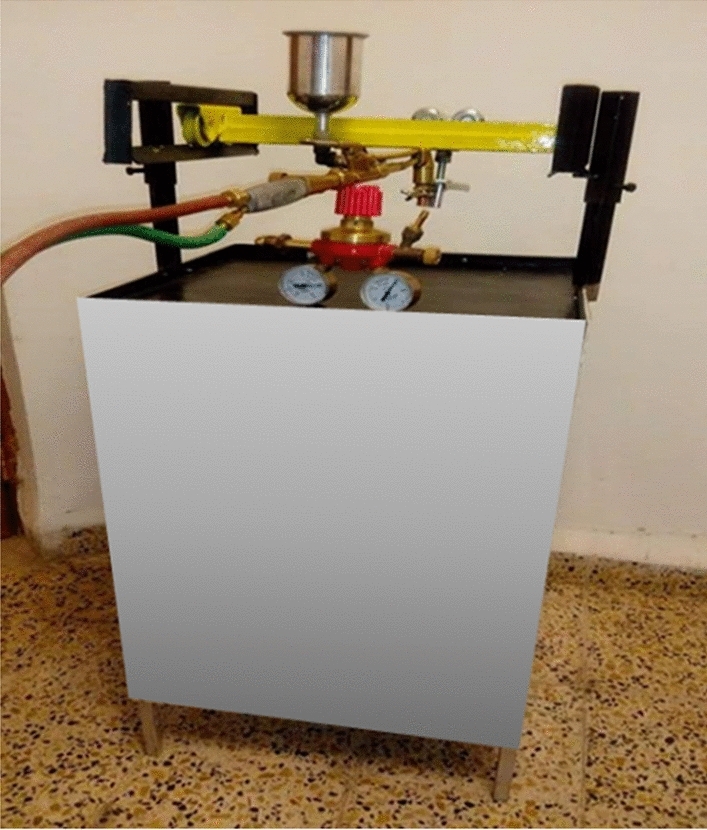
High velocity oxy-fuel system (HVOF)^15^.

High Velocity Oxy-Fuel (HVOF) is used for metallic and non-metallic (ceramic) materials of the surface layer to form a coating layer for a semi-molten or molten state. Fuel oxygen and acetylene are both fed into the chamber by a gas flow rate ratio of 3:1 for oxygen and acetylene applied with a pressure of 10 bars for oxygen and 5 bars for acetylene. Both gases and nanoparticles are controlled by special valves^14^, shown in Fig. 2. The feeding rate of tungsten carbide nanoparticles was about (1.3*10^–2^ g/s).

### Preparing the samples inside the elbow

**Figure 3:**
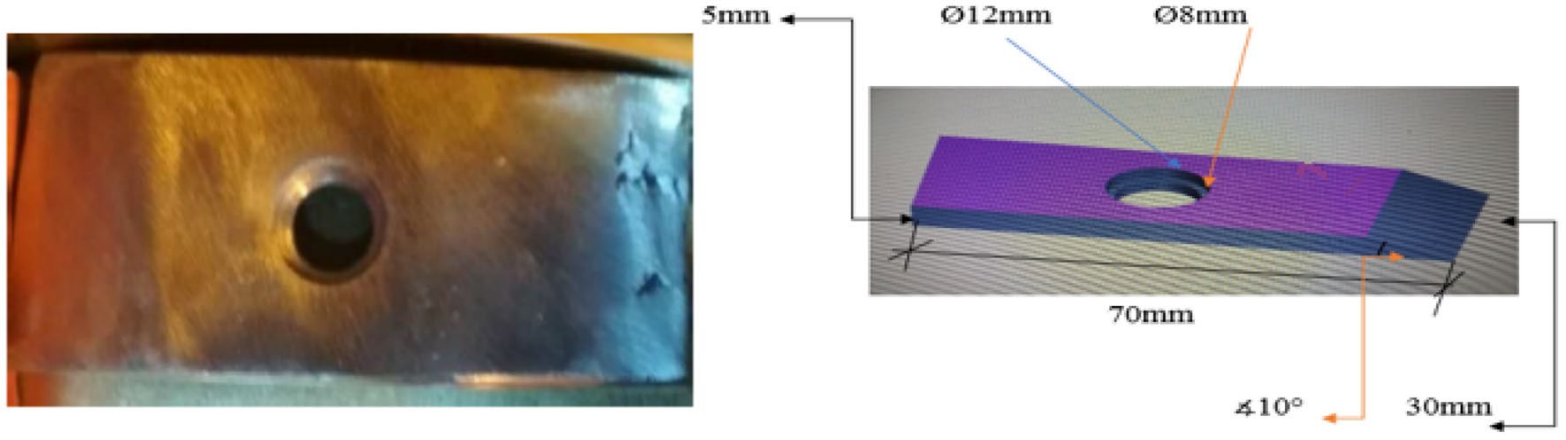
Samples inside Elbow.

The samples used to carry out the test inside the elbow prepared from low carbon steel with dimensions of 70*30*5 mm and inner surface roughness of 0.9 mm, and the location of samples the inside elbow is set to ensuring that they exposed to real harsh operating conditions and the flow is easy to run and pass through the forms to ensuring contact at every point on the surface of each sample. Furthermore, holes drilled into the sample to make it easier to install inside the elbow with the best types of fastening, shown in Fig. 3. A 10-degree cut angle created with the horizon at one side of each sample to avoid flow separation zones behind the site where the flow starts to pass over the sample. Additionally, the curvature of the surface of the samples was accurately made to make it easily fit inside the elbow.

### Samples testing inside the elbow

The steps of fixing the samples inside the elbow are as following:

1. Four holes of 8.5 mm in diameter drilled in the elbow to fixing the samples.
2. Based on the pre-simulation results, a 10-degree cut angle was made with the x-axes at the front side of each sample (as shown in Fig. 3) to prevent any expected flow separation zones behind the location where the flow starts to pass over the sample and keep the flow fully attached to the sample’s surface
3. Measuring the weight of each sample before fixing them on the inner surface of the elbow.
4. The number of samples was four from low carbon steel and fixed on the inner surface of the elbow.
5. The samples preparation were as following: one sample without coating, one sample with a coating thickness of 30 μm, one sample with a coating thickness of 40 μm, and one sample with a coating thickness of 50 μm with surface roughness (0.03–0.045 mm).

**Figure 4:**
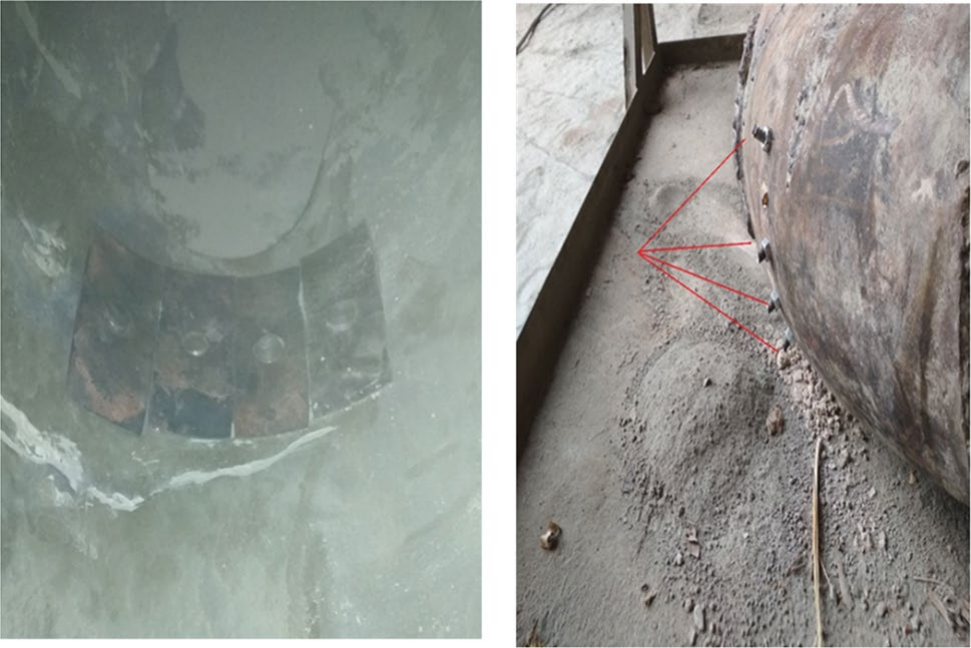
Samples installation inside the elbow.

The site chosen in the inner surface of the elbow, which is expected to subject to the most severe conditions by cement particles and flow velocity ; and the samples placed on the inner circumference of the elbow using steel bolts, shown in Fig. 4.

### Preparation of Wear test (Pin on Disc) Samples

**Figure 5:**
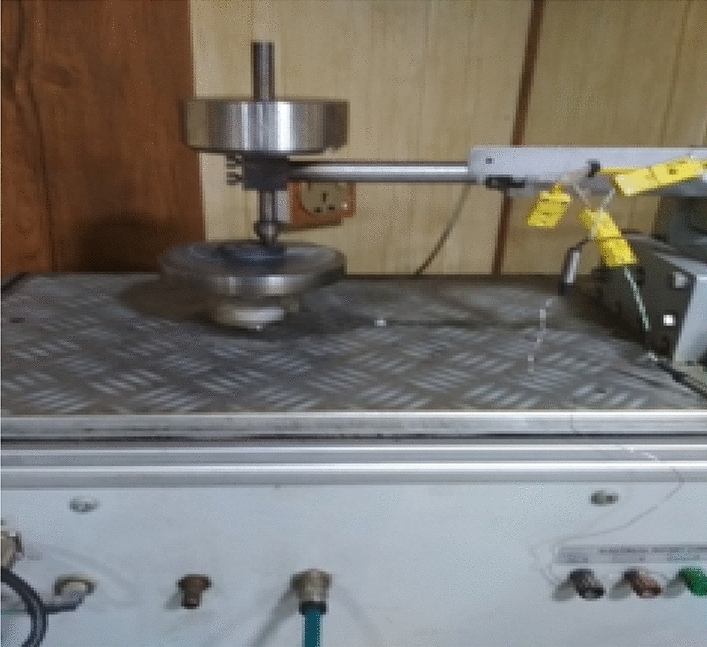
Pin on disc tester.

**Figure 6:**
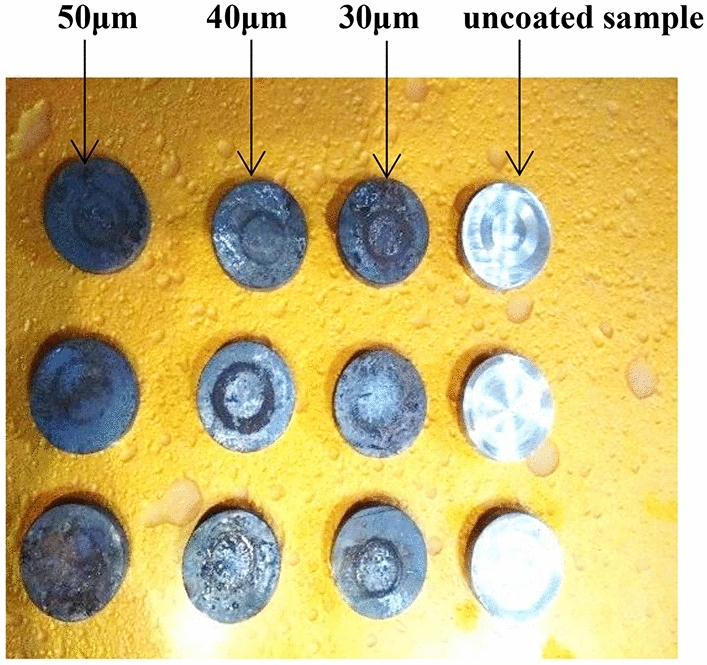
Wear samples test after testing (Coated and uncoated).

The samples preparing for wear test (pin on disc) from low carbon steel of diameter (40 mm) and thickness of (10 mm) according to the standard specification of (ASTM G-99-17)^16^. The surface of samples smoothed in the laboratories of the faculty of Engineering at the University of Kufa/ IRAQ. The pin rotation diameter is chosen according to the rotating speed (400, 500 r.p.m.) and the applied load on the pin is 20 N according to the standard specification, the abrasive wear occurs by mechanism of (deformation and cutting) for the tested samples surface by the pin from hard metal and abrasive particles will be removed continuously due to the high rotation speed as shown in Figs. 5 and 6.

## Mathmatical and numerical formulation

### Governing equations and particles tracking method

The first step in predicting erosion is the flow field simulation. Fluid flow modeled by time-averaged Navier–Stokes equations to calculate the flow fields presented. RANS method was used to model the turbulent flow. Airflow assumed incompressible, Newtonian fluid with constant fluid properties, three-dimensional, unsteady, and in turbulence mode. Hence, the continuity, momentum, and turbulence equations for an incompressible fluid, three-dimensional, and time-dependent are^17^:

Continuity equation:

1 $$\frac{\partial{\varvec{\rho}}}{\partial {\varvec{t}}}+\nabla .\left({\varvec{\rho}}\overrightarrow{{\varvec{U}}}\right)=0$$

- Momentum equations:
  2 $$\frac{\partial }{\partial {\varvec{t}}}\left({\varvec{\rho}}\overrightarrow{{\varvec{U}}}\right)+\nabla .\left({\varvec{\rho}}\overrightarrow{{\varvec{U}}} \otimes \overrightarrow{{\varvec{U}}}\right)=-\nabla .{\varvec{P}}+\nabla .\left[\left({\varvec{\mu}}+{{\varvec{\mu}}}_{{\varvec{t}}}\right)\left(\nabla .\overrightarrow{{\varvec{U}}}+(\nabla .\overrightarrow{{\varvec{U}}}{)}^{{\varvec{T}}}\right)\right]-\frac{2}{3}\nabla .\rho {\varvec{k}}{{\varvec{\delta}}}_{{\varvec{i}}{\varvec{j}}}$$

where: U is the time-averaged speed and ${\varvec{p}}$ is the pressure.

In order to find the turbulent eddy viscosity (${{\varvec{\mu}}}_{{\varvec{t}}}$), the Menter k–${\varvec{\omega}}$ SST turbulent model adopted in the current study as follows^18^:

3 $$\frac{\partial }{\partial {\varvec{t}}}\left({\varvec{\rho}}{\varvec{k}}\right)+\nabla .\left({\varvec{\rho}}{\varvec{k}}\overrightarrow{{\varvec{U}}}\right)=\nabla .\left[\left({\varvec{\mu}}+\frac{{{\varvec{\mu}}}_{{\varvec{t}}}}{{{\varvec{\sigma}}}_{{\varvec{k}}}}\right)\nabla .{\varvec{k}}\right]+{{\varvec{P}}}_{{\varvec{k}}}-{{\varvec{\beta}}}_{1}{\varvec{\rho}}{\varvec{k}}{\varvec{\omega}}$$

$$\frac{\partial }{\partial {\varvec{t}}}\left({\varvec{\rho}}{\varvec{\omega}}\right)+\nabla .\left({\varvec{\rho}}{\varvec{\omega}}\overrightarrow{{\varvec{U}}}\right)=\nabla .\left[\left({\varvec{\mu}}+\frac{{{\varvec{\mu}}}_{{\varvec{t}}}}{{{\varvec{\sigma}}}_{{\varvec{\omega}}1}}\right)\nabla .{\varvec{\omega}}\right]+{\varvec{\gamma}}\left({2{\varvec{\rho}}{\varvec{\delta}}}_{{\varvec{i}}{\varvec{j}}}.{{\varvec{\delta}}}_{{\varvec{i}}{\varvec{j}}}-\frac{2}{3}{\varvec{\rho}}{\varvec{\omega}}{\nabla .\overrightarrow{{\varvec{U}}}{\varvec{\delta}}}_{{\varvec{i}}{\varvec{j}}}\right)$$

4 $$-{{\varvec{\beta}}}_{2}{\varvec{\rho}}{{\varvec{\omega}}}^{2}+2\frac{{\varvec{\rho}}}{{{\varvec{\sigma}}}_{{\varvec{\omega}}2}{\varvec{\omega}}}\nabla .({\varvec{k}}.{\varvec{\omega}})$$

where: $${{\varvec{P}}}_{{\varvec{k}}}=2{{{\varvec{\mu}}}_{{\varvec{t}}}{\varvec{\delta}}}_{{\varvec{i}}{\varvec{j}}}.{{\varvec{\delta}}}_{{\varvec{i}}{\varvec{j}}}-\frac{2}{3}{\varvec{\rho}}{\varvec{k}}{\nabla .\overrightarrow{{\varvec{U}}}{\varvec{\delta}}}_{{\varvec{i}}{\varvec{j}}}$$ and $${{\varvec{\delta}}}_{{\varvec{i}}{\varvec{j}}}=\frac{1}{2}\left(\frac{\partial {{\varvec{u}}}_{{\varvec{i}}}}{\partial {{\varvec{x}}}_{{\varvec{j}}}}+\frac{\partial {{\varvec{u}}}_{{\varvec{j}}}}{\partial {{\varvec{x}}}_{{\varvec{i}}}}\right)$$

Thus, the turbulent eddy viscosity can calculated from ${{\varvec{\mu}}}_{{\varvec{t}}}={\varvec{\rho}}{\varvec{k}}/{\varvec{\omega}}$

The empirical constants and Pr_t_ presented in the above equations can set to the following values^18^:

$${{\varvec{\sigma}}}_{{\varvec{k}}}=1.0$$, $${{\varvec{\sigma}}}_{{\varvec{\omega}}1}=2.0$$, $${{\varvec{\sigma}}}_{{\varvec{\omega}}2}=1.17$$, $${\varvec{\gamma}}=0.44$$, $${{\varvec{\beta}}}_{1}=0.09$$, $${{\varvec{\beta}}}_{2}=0.083$$, $${{\varvec{P}}{\varvec{r}}}_{{\varvec{t}}}=0.9$$

Once the simulation of the flow field has been completed, the particle tracking technique applied. In this technique, STAR CCM^+^ CFD models motion of particles as a discrete phase (solid particle) in a Lagrangian reference frame and calculates the trajectories of these particles using Newton’s second law of motion (equation of particle motion) on an individual scale. The particle momentum can formulate as^18^;

5 $${{\varvec{m}}}_{{\varvec{p}}}\frac{{\varvec{d}}{{\varvec{u}}}_{{\varvec{p}}}}{{\varvec{d}}{\varvec{t}}}={{\varvec{F}}}_{{\varvec{D}}}+{{\varvec{F}}}_{{\varvec{G}}}+{\varvec{F}}$$

where: ${{\varvec{m}}}_{{\varvec{p}}}$, ${{\varvec{u}}}_{{\varvec{p}}}$ and t are mass of particle in kg, particle speed in m/s, and time in second, respectively. ${{\varvec{F}}}_{{\varvec{D}}}$ Represents the drag force acting on the particle in N, and ${{\varvec{F}}}_{{\varvec{G}}}$ represents the gravitational force in N. Any other external forces are included in F.

The drag force can compute as;

6 $${{\varvec{F}}}_{{\varvec{D}}}=\left(\frac{1}{{{\varvec{\tau}}}_{{\varvec{p}}}}\right){{\varvec{m}}}_{{\varvec{p}}}\left({\varvec{u}}-{{\varvec{u}}}_{{\varvec{p}}}\right)$$

where ${{\varvec{\tau}}}_{{\varvec{p}}}$ the response time of particle speed , u is the instantaneous flow velocity which can calculated from ${\varvec{u}}={\varvec{U}}+\bar{u^\prime}$ where U is the time-averaged velocity calculated directly from Eq. (4) and $\bar{u^\prime}$ is the turbulent fluctuation speed, which defined as;

7 $$\bar{u^\prime}=\sqrt{\frac{2k}{3}} $$

where: k is the turbulent kinetic energy.

The gravitational force formulated as;

8 $${F}_{g}={m}_{p}g\frac{\left({\rho }_{p}-\rho \right)}{{\rho }_{p}}$$

where: ${\rho }_{p}$ is density of the particle, ρ is the density of the fluid, and g is the gravitational direction.

### Erosion model

Erosion modeling predicts the rate of erosion from particle impact on solid boundaries. In calculating the erosion rate, STAR-CCM + accumulates the damage that each particle impacts cause. This calculation is done by selecting a correlation for the erosion ratio, that is, the mass eroded from the wall per unit mass of impinging particles. Erosion rate is computed by accumulating the damage on contact boundary faces that each solid particle impact does on the face^19^:

9 $${E}_{f}=\frac{1}{{A}_{f}}{\sum }_{\pi (f)}{\dot{m}}_{\pi }{e}_{r}$$

where ${A}_{f}$ is the area of each face, ${\dot{m}}_{\pi }$ is the particles mass flow rate in parcel $\pi$, and ${e}_{r}$ is the erosion ratio. Different correlations are available in the open literature to model the erosion rate. In the present study, the erosion ratio ${e}_{r}$ has been computed using the following Oka correlation.

#### Oka correlation

The Oka correlation for the erosion ratio is^19,20^

Where:

10 $${\mathrm{e}}_{\mathrm{r}}={\mathrm{e}}_{90}\mathrm{g}\left(\mathrm{\alpha }\right){\left(\frac{{\mathrm{v}}_{\mathrm{rel}}}{{\mathrm{v}}_{\mathrm{ref}}}\right)}^{{\mathrm{k}}_{2}}{\left(\frac{{\mathrm{D}}_{\mathrm{p}}}{{\mathrm{D}}_{\mathrm{ref}}}\right)}^{{\mathrm{k}}_{3}}$$

• The angle function $\mathrm{g}(\mathrm{\alpha })$ is defined as:

$$\mathrm{g}\left(\mathrm{\alpha }\right)={{(\mathrm{sin\alpha })}^{{\mathrm{n}}_{1}}(1+{\mathrm{H}}_{\mathrm{v}}(1-\mathrm{sin\alpha }))}^{{\mathrm{n}}_{2}}$$

• $${\mathrm{n}}_{1}$$, $${\mathrm{n}}_{2}$$ are constants.

• ${\mathrm{H}}_{\mathrm{v}}$ is the Vickers hardness of the removed material in units of GPa.

•${\mathrm{v}}_{\mathrm{rel}}$ is the relative velocity of the solid particle with respect to the contact boundaries, $\left|{\mathrm{v}}_{\mathrm{rel}}\right|, \left({\mathrm{v}}_{\mathrm{rel}}={\mathrm{v}}_{\mathrm{particle}}-{\mathrm{v}}_{\mathrm{wall}}\right)$.

•${\mathrm{v}}_{\mathrm{ref}}$ is the reference velocity.

•${\mathrm{D}}_{\mathrm{ref}}$ is the reference diameter.

•${\mathrm{k}}_{2}$ and ${\mathrm{k}}_{3}$ are constants.

. $${\mathrm{e}}_{90}={{\mathrm{e}}_{\mathrm{r},\mathrm{DNV}@90({\mathrm{D}}_{\mathrm{ref}}/{\mathrm{D}}_{\mathrm{p}})}}^{{\mathrm{k}}_{3}}$$

### Boundary conditions

**Figure 7:**
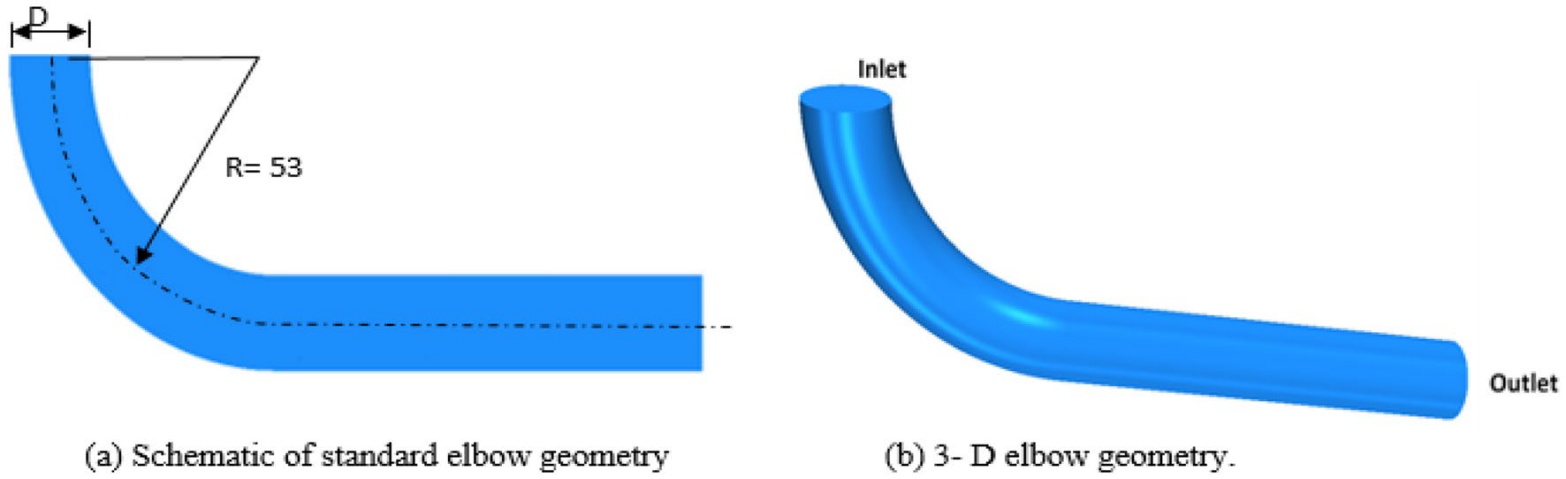
Schematic of the elbow.

Figure 7 shows the simulated geometry, which the standard elbows that have already been in use as one of the cement transport equipment et al.-Najaf cement factory. The elbow has a diameter of D = 19 cm with a curvature ratio (R/D) equal to 2.78. A constant pressure boundary condition was applied at the outlet section with a pressure equal to atmospheric pressure (101325 Pa). The initial flow and boundary conditions have been listed in Table 2.

**Table 2:** Initial boundary conditions of flow inside elbow and pipe^20^.

Parameter	Value
Inlet temperature	T air = 323 °K T cement = 393–403 °K
Carrier fluid	Air (the air properties were set at T = 323 °K)
Bulk air velocity entering elbow	21 m/s
Average Cement particle diameter	60 μm
Cement density	1600 kg/m^3^
Pipe diameter Curvature ratio(R/d)	190 mm 2.78
Pipe material	Low carbon steel

### Grid generation

**Figure 8:**
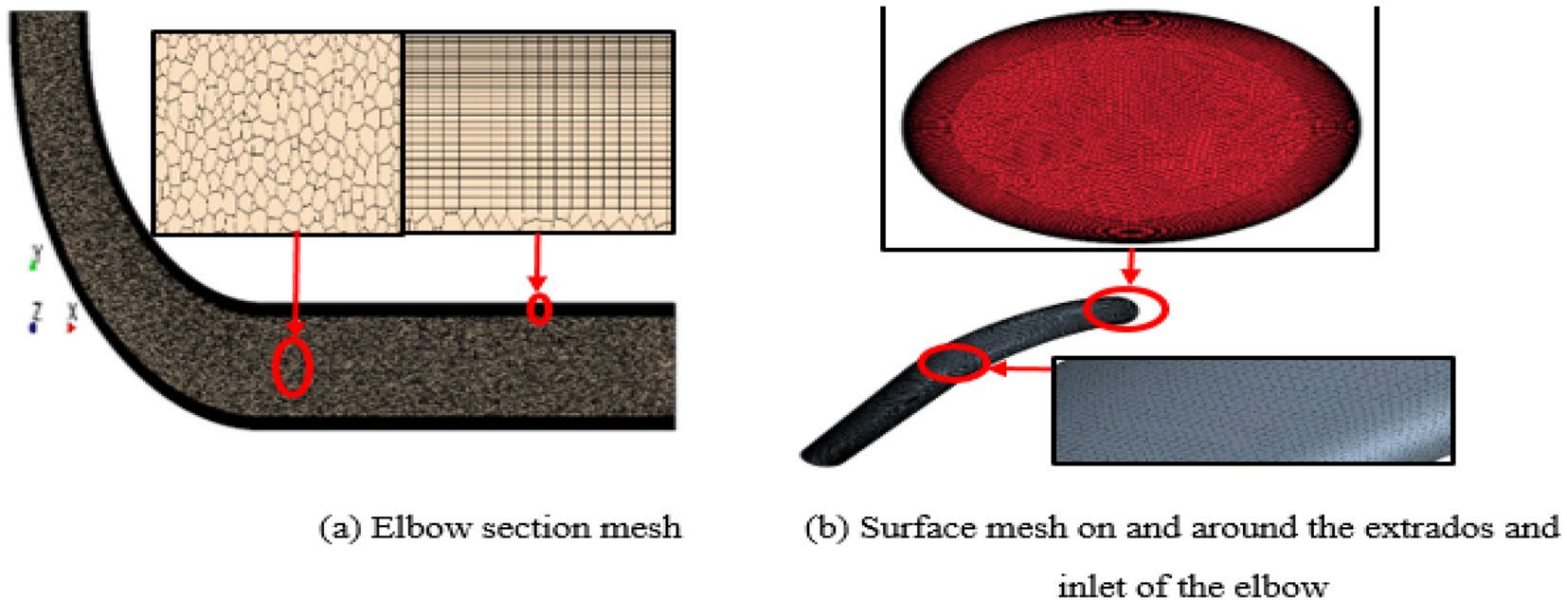
Mesh of elbows model.

A three-dimension, multi-blocks, hexahedral and polyhedral meshes were created using the mesh generation section of STAR CCM^+^ code to generate the entire computational domain of the elbow. The mesh generation structure is shown in Fig. 8a,b. Grid independence study performed using a four-mesh study to ensuring that the simulations acquired using the selected grid are independent of the grid size. Overall erosion rate used as an indicator of convergence, and the results of the grid independence test are illustrated in Table 3. Consequently, the current simulations were performed using the mesh number of (4157524) based on the overall erosion rate results obtained from the grid independence study. The surface roughness of the elbow for coated (0.038 mm) and un-coated model (0.9 mm) took into consideration in the model solution.

**Table 3:** Mesh refinement study.

Number of grids	Overall erosion rate (g/hr)
1,013,219	0.3924
2,452,810	0.4081
4,157,524	0.4106
5,609,287	0.4166

### Numerical procedure

**Figure 9:**
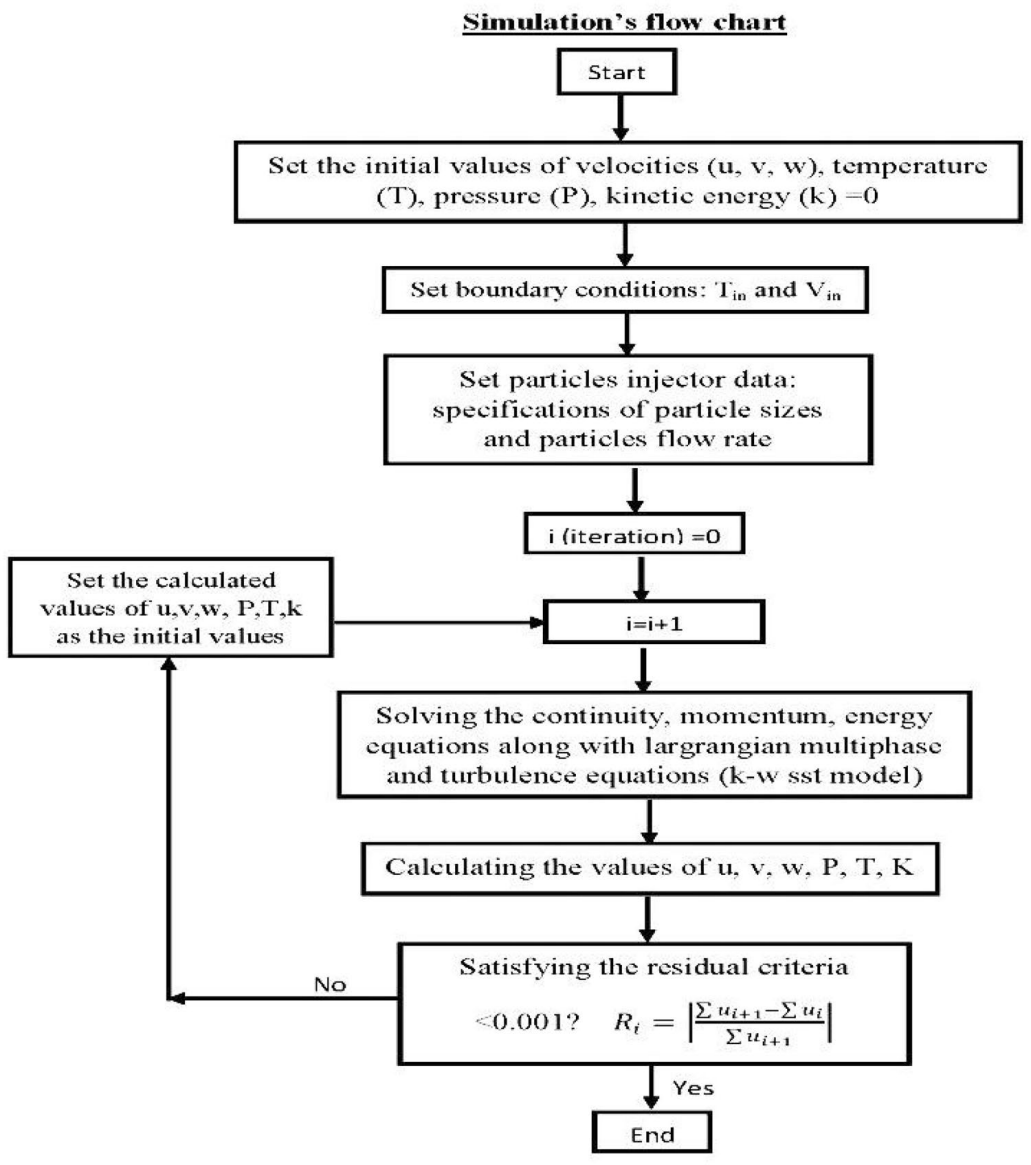
Flow chart of numerical simulation.

The Eulerian-Lagrange simulation performed within the STAR CCM + firstly solves the system governing equations discretized by the finite volume technique along with using the SIMPLE algorithm method. In conjunction with this, the Menter k– SST RANS turbulence model employed to predict the turbulence effects on the airflow field ^17^. The relaxation Gauss–Seidel scheme is applied to solve iteratively the generated set of linear algebraic equations to calculate the values of velocity, pressure, etc. A time step of ~ 1 × 10^–4^ s. was used for all calculations. Airflow field simulation was run for a 24-time cyclea to reach stable statistics results of the flow field and turbulence quantities. The solution procedure continued until reach the error criterion of for every component to satisfy the solution convergence criterion and Fig. 9 show the flow chart of numerical simulation.

## Result and disscussion

### Results of experimental work

#### Sample tested inside elbow

**Figure 10:**
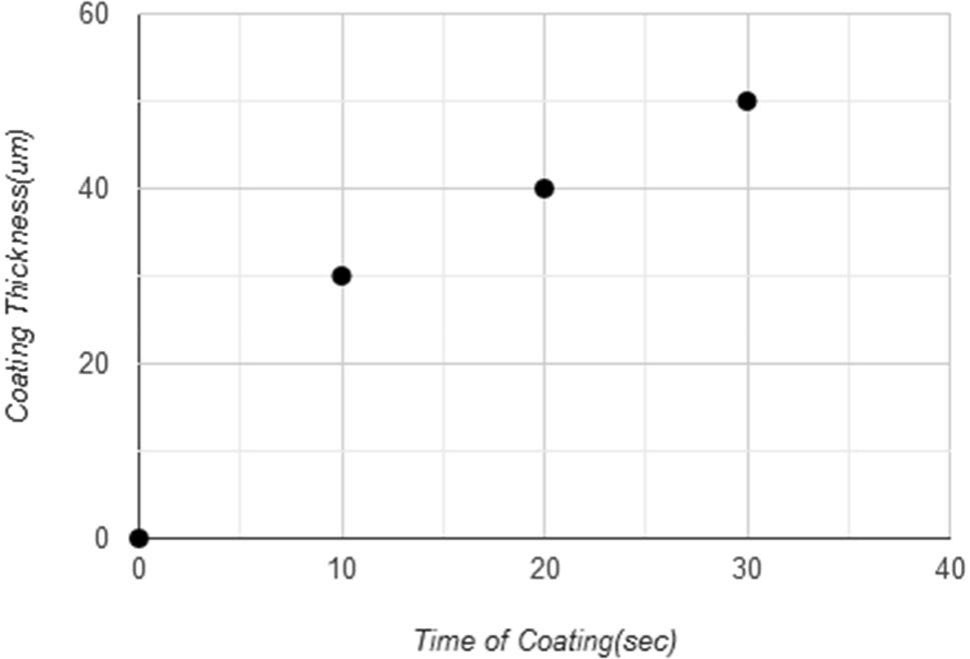
Coating layer thickness in (μm) with time of coating in (s).

**Figure 11:**
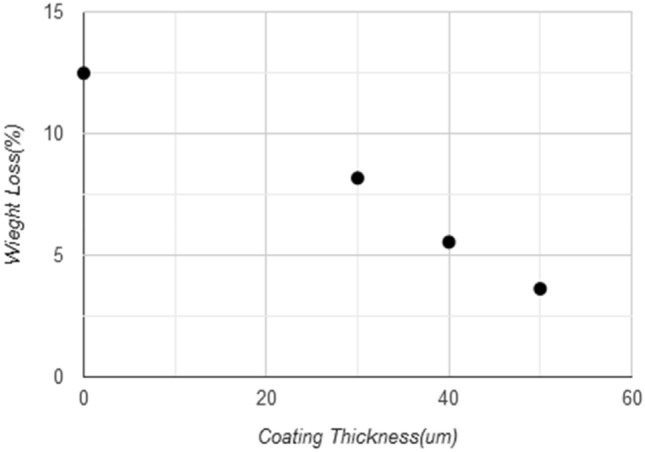
Weight loss ratio with coat thickness of samples inside elbow.

**Figure 12:**
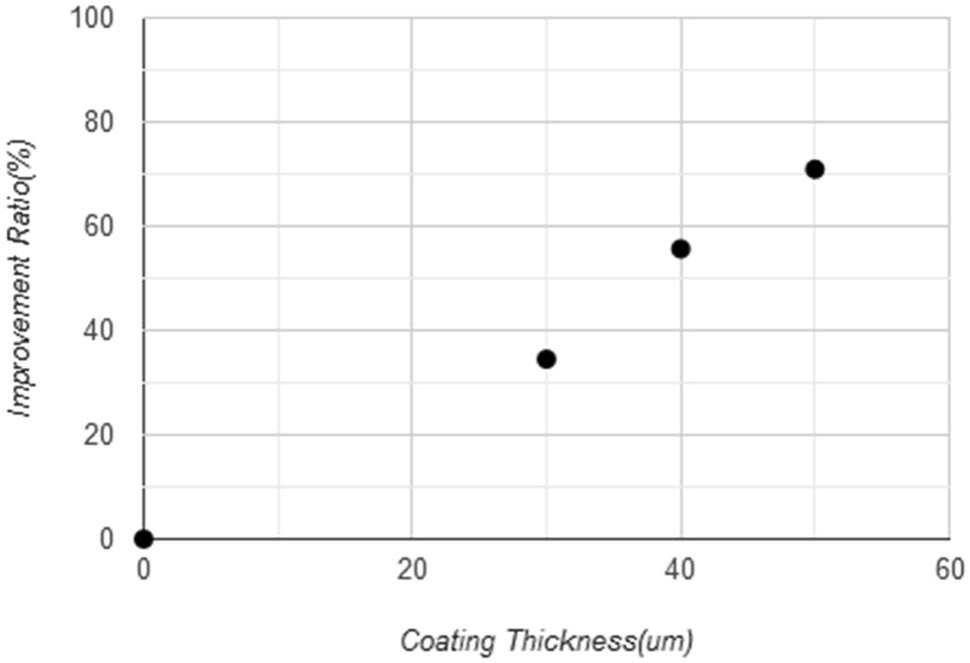
Improvement ratio of wear resistance for samples inside the elbow.

The tested samples were 4 samples, one without coating and three with different coating thicknesses 30, 40, and 50 μm which are gotten from different periods of coating time 10, 20, 30 s, while the time coating more than 30 s (more than 50 μm) will give an unstable coating layers(cracking, lacking, and failure) due to the lack of adhesion between the sample surface and coating layer as shown in Fig. 10, and that occurs due to the feeding rate of nanoparticles, the flame control, and personal ability to controlling the coating process. The operating conditions for cement transporting pipes and elbows was 30 tons/hr, and the total time was 170 working hours during which about 5100 tons were passing through the pipe. The graph shown in Fig. 11 shows the relationship between the coating thickness and weight loss ratios, where it can show that when increasing the coating thickness to 50 μm, the lowest percentage of weight loss reached to 3.62%, which indicates homogeneity, reduction of defects, the high strength and hardness of coating layer, and regular spread and high the intensity of (WC) that make elbow and pipe surface more resistant to abrasion and ablation. The improvement ratio in wear resistance for coated sample reaches 71% for the maximum coating thickness of 50 μm, as shown in Fig. 12. This coating layer plays a role as protect shields for the inside surface of the elbow toward the high-velocity cement particles that may cause erosion for the elbow surface, as this coating the layer had good adhesion properties with an elbow inside surface and good mechanical properties of tungsten carbide nanoparticles especially the hardness and wear resistance.

#### Wear test (pin on disk) results

**Figure 13:**
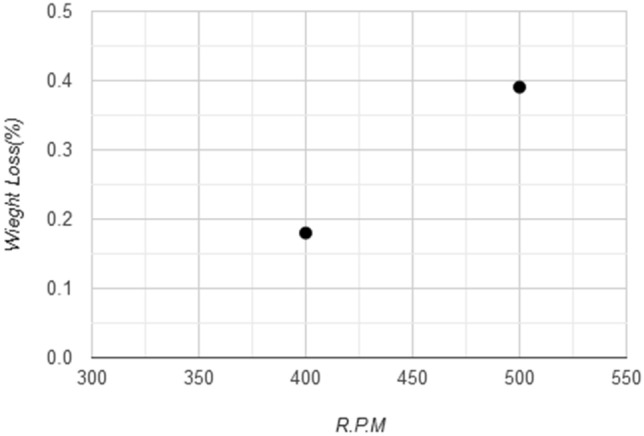
Weight loss ratio with angular velocity for uncoated samples.

Wear test based on weight loss, which was conducted on samples in the laboratory to compare between the wear resistance of coated and uncoated samples and simulate the result from the experimental test and its conformity. This test was done with (24) samples, through which a comparison made between samples without coating, (6) samples coated with a thickness of 30 μm, (6) samples coated with a thickness of 40 μm, and finally (6) samples with a 50 μm coating, the samples were weighed before the test by an exact digital balance (4 digits), The time to complete the test for each sample was (10 min). The Fig. 13 the results shows the wear resistance of samples without coating layer under conditions of (20 N) loading and angular velocity (400, 500 R.P.M) that the weight loss increase with increase rotating speed and friction and the best weight loss rate at speed (500 R.P.M) was (0.3905%).

**Figure 14:**
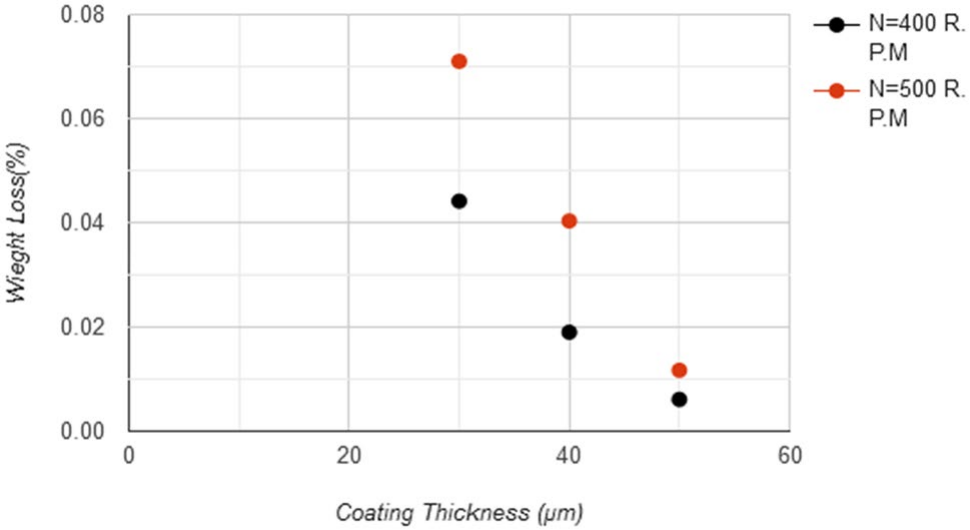
Coating layer thickness and weight loss for different rotational speed at force 20 N.

**Figure 15:**
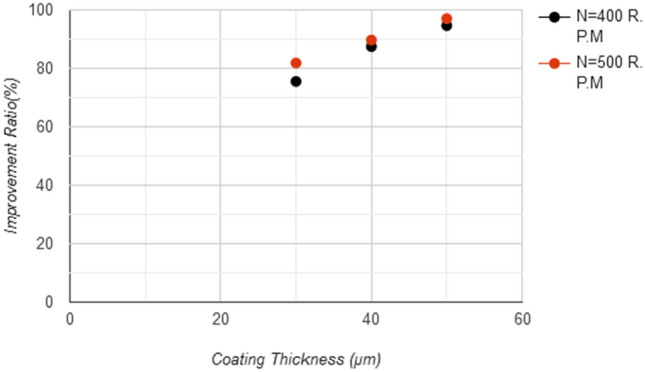
Coating layer thickness with improvement ratio in wear resistance test (pin on desk).

The Fig. 14 shows the results of the wear test(pin on disc) for samples with a coating layer thickness 30, 40, and 50 μm under conditions of (20 N) loading and angular velocity 400, 500 R.P.M., the weight loss increase with increase rotating speed and friction, the lowest the weight loss rate was at speed 500 R.P.M, for sample coated with 50 μm with value %0.0117, while Fig. 15 shows the improvement ratio for wear resistance reaches 97% for sample coated with 50 μm thickness due to the high strength, high hardness, good adhesion, and homogeneity of the Nano tungsten carbide coating layer, that makes it more resistant to erosion and penetration by hard particles.

#### Scanning electron microscope (SEM) image analysis

**Figure 16:**
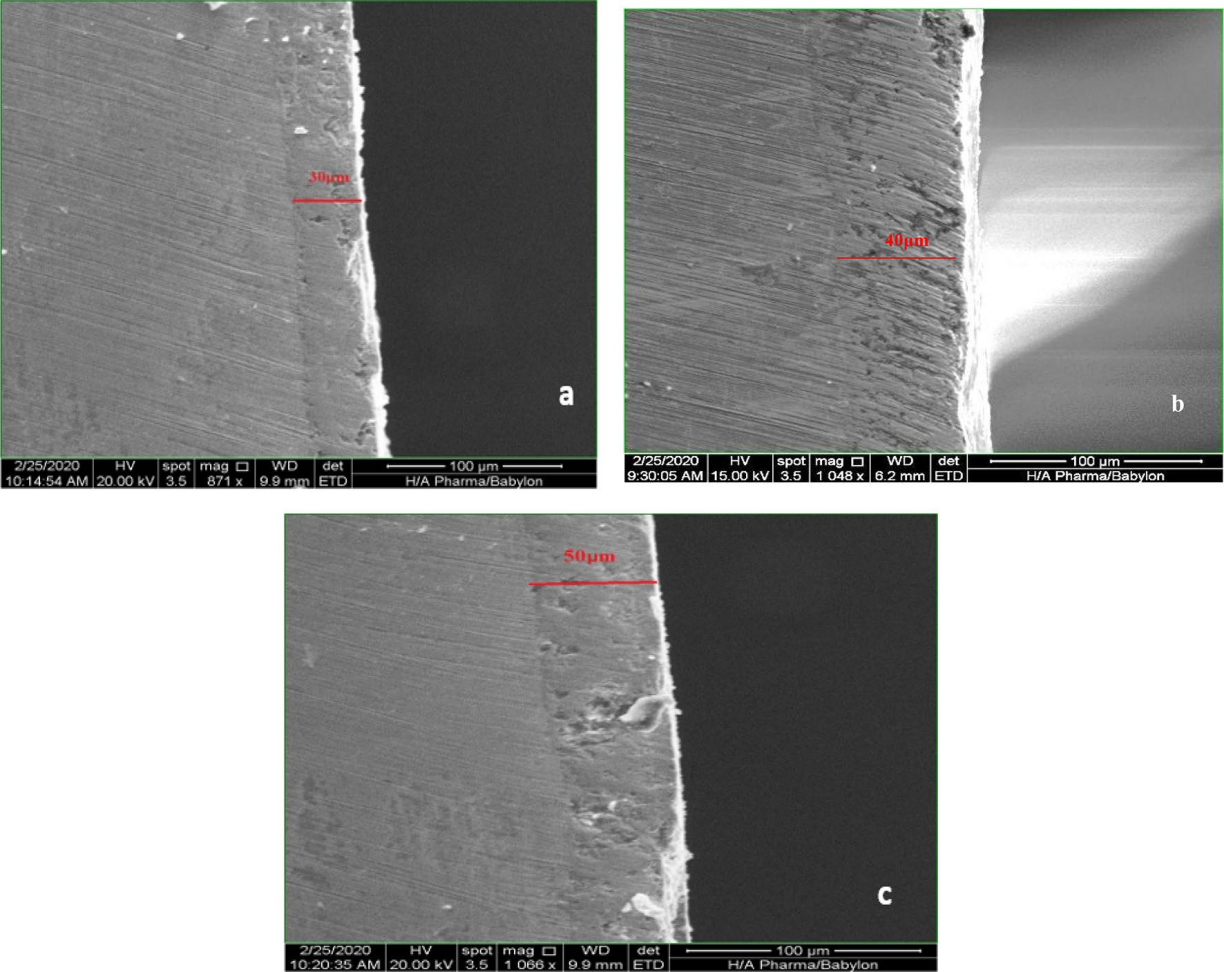
SEM images for coating thickness. (**a**) 30 µm, (**b**) 40 µm, (**c**) 50 µm^21^.

The thickness of the coating layer of the samples was calculated at different coating times by SEM Images and the measurement of the coating thickness was done by the image J soft program. It was found that the layers thicknesses of 30, 40, and 50 µm were achieved for coating times of 10, 20, and 30 s, respectively as shown in Fig. 16.

**Figure 17:**
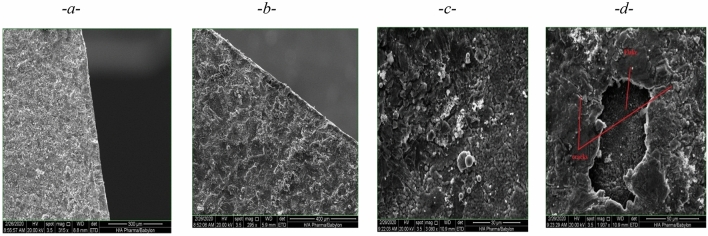
(**a**) 30 µm, (**b**) 40 µm, (**c**) 50 µm, (**d**) thickness more than 50 μm (Cracking, Flacking, and failure of coating for coating time more than 30 s).

The SEM images showed also the coating time more than 30 s (thickness layer more than 50 μm) will produce an unstable coating layer that subjected to cracking, flacking, and failure due to lack of adhesion between the sample surface and coating layer as shown in Fig. 17 comparison with stable coating layers of thickness 30, 40, and 50 μm.

### Results of numerical simulation

**Figure 18:**
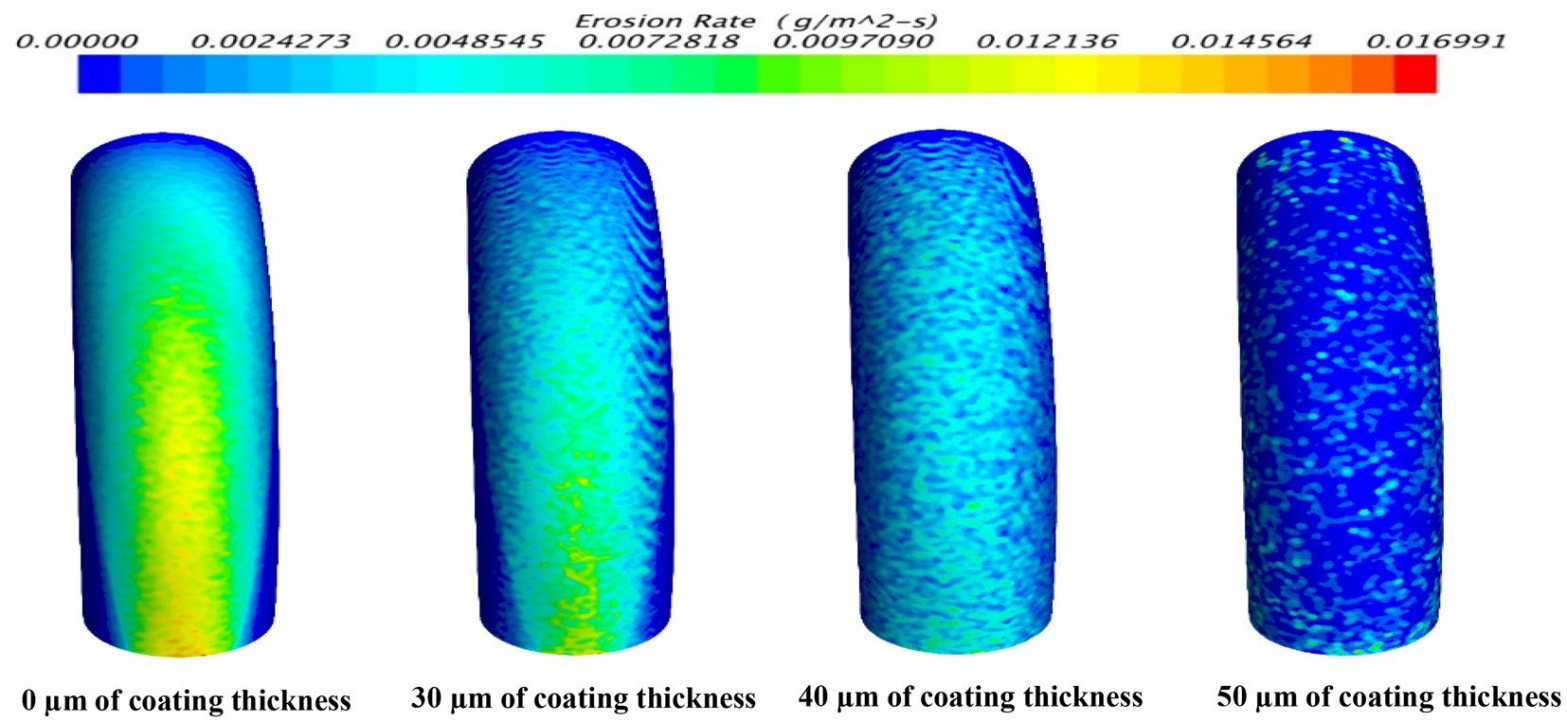
The distributions of the erosion rate profile for the elbow with different values of coating thickness.

Figure 18 presents the predicted erosion profile distribution for the inner surface of the elbow with different thicknesses of coating layer as a simulation for the real operation test. In this simulation, Oka Correlation is used to model the erosion rate. It has been seen that the erosion rate is reduced by increasing the coating thickness of tungsten carbides nanoparticles, as this coating layer, protect the inner surface of the elbow from the friction with high-velocity cement particles. This coating layer plays a role as protecting shields the inside surface of the elbow toward the high-velocity cement particles that may cause erosion for the elbow surface , this coating layer had good adhesion properties with an elbow inside surface and good mechanical properties of WC nanoparticles especially hardness and wear resistance.

**Figure 19:**
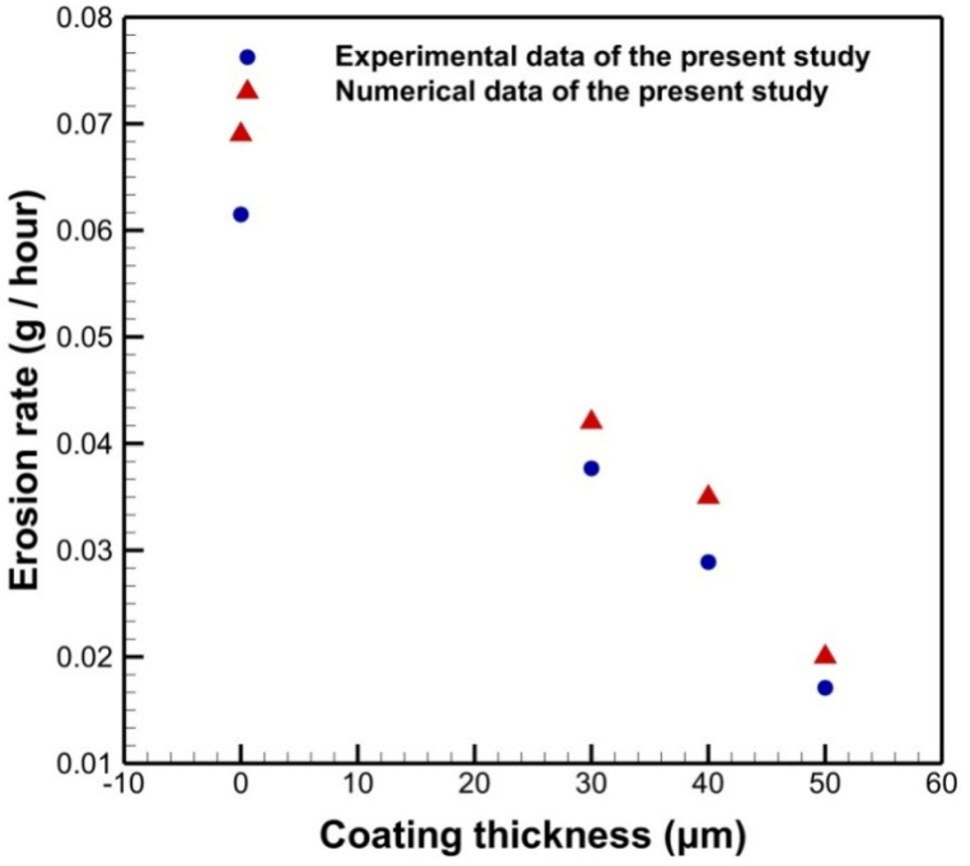
Comparison between the experiment and numerical data for Erosion rates for different coat thickness.

Figure 19 illustrated validation of the predicted erosion rate with that measured in the experimental work. The erosion rate is calculated in (g/hr) per wall surface area of (7 * 5 cm). This figure shows a good agreement with the experimental results although a slight disagreement is visible. This mismatch is due to the numerical accuracy and the inlet boundary condition especially the inlet speed which cannot be measured accurately from the cement factory due to measurement difficulties. The figure showed the agreement in erosion rate between the numerical and experimental results for test samples inside the elbow under the same operational conditions.

## Conclusions

The failure due to mechanical wear in pipes and elbows may cause a lot of losses in money and times for industrial foundations and this failure occur due to ***Erosion*** that involves the removal of material from the surface of a component by the high-speed impact of a liquid or of a stream of hard particles carried in a fluid flow. The mechanism of erosion occurs when a stream of hard particles is directed at a surface. This may be intended, as in shot blasting processes, or it may arise incidentally, such as in pipelines and associated components carrying slurries or crude oil containing sand same as pipes and elbows transporting cement powder. This work aimed to find a unique technique to reducing this defect by using Nanoparticle coating with tungsten carbides (WC). This coating technique gave an improvement in wear resistance towards the cement particles flow by 71% for the sample with 50 μm coating thickness that tested inside elbow under same real operation conditions to get a vision about the behavior of coated and uncoated samples inside the elbow is facing the high-velocity cement particles flow. The coating layers can recoat after its removal many times. Also, the improvement in wear resistance for the 50 μm coating thickness sample that was tested by a specific standard test of wear (pin on disc) was 97% higher than samples without a coating layer. The result of the pin on disc test indicates the effect of the Nano coat layer which prevents the steel surface from degradation and increasing the operating life of pipes and elbows. The coating layer thickness more than 50 μm will subject to cracking, flacking, and failure due to the lack of adhesion with the steel surface. The numerical simulation that was done to compare with the test inside the elbow shows a good agreement between the experimental results and numerical results with a convergent reach to 90%.
